# Soft Tissue Mandibula and Tongue Reconstruction Using A Suprafascial, Folded, Deepithelialized Antero-Lateral Thigh Perforator Free Flap

**DOI:** 10.29252/wjps.8.1.103

**Published:** 2019-01

**Authors:** Christian Weinand, Carsten Dittes

**Affiliations:** 1 Department of Plastic and Reconstructive Surgery, Hand Surgery, Dietrich-Bonhoeffer-Klinikum Neubrandenburg, Germany;; 2 Department of Oral- Maxillo-Facial Surgery, Plastic Operations, Dietrich-Bonhoeffer-Klinikum Neubrandenburg, Germany

**Keywords:** Flap, Mandible, Tongue, Reconstruction, Sqamous cell carcinoma, Surgery

## Abstract

Head and neck squamous cell carcinoma (HNSCC) is the most frequent carcinoma of the head and neck region. For coverage of an entire resected mandible with floor of the mouth, 3/4 of the tongue and soft tissue of cheeks and neck bony reconstruction of the mandible and soft tissue reconstruction of tongue, cheeks and a neck large flap are needed. A patient with a superinfected T4 HNSCC was presented to our outpatient clinic. Complete resection of the mandible, bilateral neck dissection and 3/4 resection of the tongue were performed. A complex reconstruction using two free flaps was not feasable, so a large, folded, suprafascial Antero Lateral Thigh Perforator (ALTP) flap for immediate soft tissue reconstruction was used. Because of the anatomy, no reconstruction plate was inserted. On postop day 11, an understandable speaking was possible using a speach canula. Swallowing was possible without regurgitation. Eight months postoperatively, the patients mimic and closure of the mouth were satisfactory. The flap was viable throughout the entire time. It was shown that the suprafascial ALTP flap was a versatile part in the armamentarium for complex mandible soft tissue reconstruction.

## INTRODUCTION

Patients with head and neck squamous cell carcinoma (HNSCC) of the oral cavity often present at a late stage with lymph node metastasis. Abuse of tobacco, alcohol consumption, Bethel nut chewing and infection with high-risk types of human papillomavirus (HPV) increases the risk of developing HNSCC.^1^ In late stages of HNSCC such as T3 or T4, the gold standard of treatment nowadays has been combined surgery and adjuvant radiation therapy and/or chemotherapy. However, surgery resulst in large facial defects and outcomes are generally poor (20% five-year survival rates).^1^

The Antero Lateral Thigh Perforator (ALTP) flap represents the workhorse for non osseous components in reconstructive surgery.^2^^,^^3^ Especially, Wei *et al.* have instituted the ALTP flap for reconstruction of head and neck defects.^2^^,^^3^ However, the use of the ALTP flap for soft tissue reconstruction of the mandible and floor of the mouth has been described rarely. We present the use of a large suprafascial ALTP flap, folded and partially deepithelialized for defect coverage of a completely resected mandible and reconstruction of the floor of the mouth and 3/4 of the tongue in a patient with pT4 HNSCC.

## CASE REPORT

**Fig. 1:**
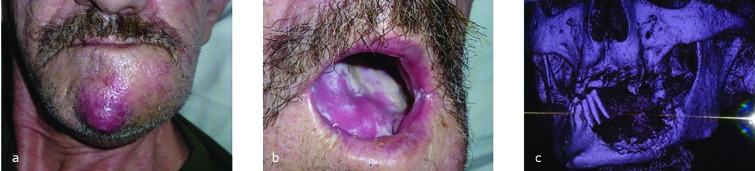
a, b, c: Clinical appearance and dental CT of the patient

A 56-year old patient was referred to our outpatient clinic for squamous cell carcinoma of the floor of the mouth. Figure 1 shows the clinical appearance with cutaneous metastasis of the squamous cell carcinoma (a) and intraoral leukoplakia (b). Magnetic Resonance Imaging (MRI) showed a squamous cell carcinoma transversing the enoral middle line ventrally, infiltration of tongue muscle and mandibular bone left and paramedian with infiltration into the lower lip and into the subcutanuous fat of the chin. Necrotic cervical lymphatic nodes level Ib and II were described. Dental Computertomography (CT) (Figure 1c) revealed large osteonecrosis of the mandible with a pathological dislocated fracture of the left ramus. 

**Fig. 2:**
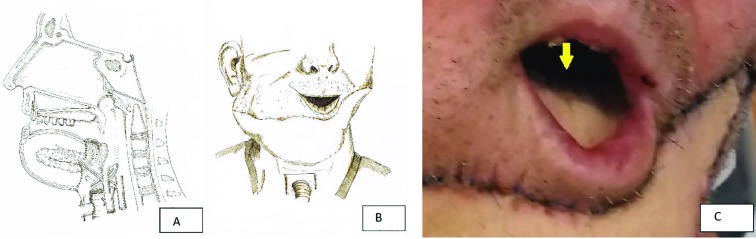
a, b, c: Illustration of the surgical reconstruction and clinical appearance. The arrow points at the remaining base of the tongue

Figure 2 is the dental CT, revealing a pathological fracture of the left ramus of the mandible and significant loss of teeth. No further metastases were located. The patient was graded pT4a, pN2c (4/ 34), M0, G2, L0, V1, stage IVa (UICC). Our tumor board in conjunction with the commitee for ethics (IRB) of our hospital advised surgical resection and radio/chemotherapy. The main goal was to achive a locoregional disease free time of one year after complete surgical resection and then prosecute on a two stage approch for reconstruction: immediate reconstruction of the soft tissue defect, second after one-year disease free time reconstruction of the mandible. After implanting a port system and a PEG, angiography excluded hemodynamically significant stenosis in both carotid arteries. 

A supraomohyoidale neck dissection on the right, a radical neck dissection on the left, a 3/4 resection of the tongue, a complete resection of the mouth floorwas was performed. The lower lip with its orbicular muscle could be preserved. Soft tissue defect size was 351 cm². An ALTP flap from the left thigh sized 28x14 cm was harvested. Primary closure of the defect was possible. Left external carotid artery and vein were used for end- to side anastomosis. The flap was folded along its length axis simulating the mandible shape sutured to itself and flap sutured to the skin. 

The enoral part of the flap was sutured to the remaining of the tongue, the remaining lower lip onto a part of the flap, that was deepithelialized in shape of the remaining lower lip. No reconstruction plate was used to not compromise the vascular supply of the anastomosis, because the supplying vessel inserted at the flap at hight of a reconstruction plate, where it would have been osteosynthesized to the remaning mandible. Last, a tracheostomy was performed. Figure 2 shows an illustration imaging of the surgical reconstruction of the cutaneous mandible, tongue and illustrating arterial and venous anastomosis (a) and the clinical appearance (b), the flap was sutured to the remaining base of the tongue, reconstructing the floor of the mouth (c). The flap healed without complications and was completely viable. Speach physiotherapists trained speach and swallowing twice per week for the patient. A speach canula was used during the length of the hospital stay. 

**Fig. 3:**
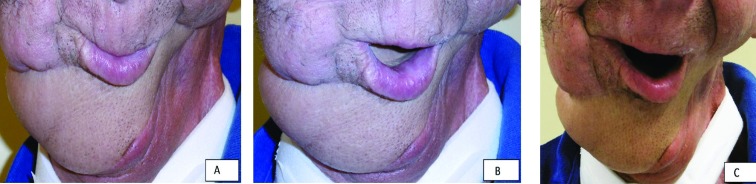
a, b, c: Clinical results 8 months, postoperatively

Histopathological evaluation confirmed R0 resection status. On day 11 postoperatively, understandable communication was possible. Mimics and motoric function of the face and remaining mandible were acceptable at time of discharge. After radiochemotherapy, the flap was still viable, no dehiscence of the scars was present. At 2 months postoperatively, the patient was able to communicate understandably without speach canula. Oral nourishment, glutunation and swallowing were possible without regurgiation. The patient was seen last in the outpatient clinic 8 months postoperatively. No locoregional recurrence was detected on clinical examination and on MRI scanning. Figure 3 shows the patients clinical examination 8 months postoperatively, closure of the mouth (a), saying the letter ‘O’ (b) and smiling (c). 

## DISCUSSION

After resection of composite tumors and radio/chemotherapy patients with advanced-stage HNSCC remain disease-free for 3 years in 35 to 55%. However, in 30% to 40% of patients, locoregional recurrences are discovered and distant metastases occur in 20% to 30% of HNSCCs.^4^ Because our patient presented at an advanced stage with a suprainfected pT4, N2c, M0, G2, L0, V1 HNSCC, surgical resection combined with radio/chemotherapy was advised. Eight months after surgery, clinical examination and MRI analysis did no show any local tumor recurrence. 

Because surgical resections of advanced-stage HNSCC result in disfiguring large soft tissue defects, free flaps are considered state of the art.^5^ Especially defects of tongue and pharynx affect most of speech and swallowing.^6^ Recommendations today are that defects of 50-70% of the tongue should be reconstructed with a free flap.^7^ Reconstructed tongues can mainly be used as contact enhancer to the palate for food propulsion, however movements of native tongue can never be reproduced.^8^ Although combined flaps are possible, the patients` condition and the anatomy of the supplying arteries made this possibility too risky. We decided therefore to reconstruct the patients tongue using the folded suprafascial ALTP flap folded, so it could be sutured to the base of the tongue. Thereby, a food propulsion was made possible. The patient demonstrated this from day 11, postoperatively.

Many free flaps are described for composite head and neck reconstruction.^2^^,^^3^^,^^9^ With the development of perforator flaps, the ALTP gained acceptance due to Wei *et al.*, who propagated the flap as ideal soft tissue flap in head and neck surgery, however, mainly for soft tissue reconstruction of cheek and lower lip.^2^^,^^3^ However, defects described before were limited to one cheek and parts of the mandible instead of the entire mandible, floor of the mouth and soft tissue of both cheeks and the neck. In our case, the defect comprised of the entire mandibula of 21 cm, the surrounding soft tissue with cheeks, soft tissue of the neck and 3/4 of the tongue, sizing 351 cm². 

The coverage was challenging because of the size of the defect and additionally the patient presented in a reduced state with scars over his chest, so a pectoral turn over flap or defect coverage using a complicated chimeric flap was not possible. We decided therefore, on a two stage reconstruction, using first a large suprafascial ALTP flap for soft tissue coverage, that should be folded along its axis to mimic the contours of the mandible. The best flap for bony reconstruction of the mandible is the free fibular flap.^10^ However, because the right external carotid artery was resected, only one side of the mandibular vessels were availabe for vascular flap supply. 

A one step immediate reconstruction using a free fibula was discussed, but because a radiochemotherapy was planned directly after surgery, the risk of osseus non-healing of the free fibula outweight the direct reconstruction.^11^ Hildalgo *et al.* have shown in their long term study of reconstructed mandibles that a two step approach is a viable option.^12^ A reconstruction plate could not be used because the pedicle and the anastomosis would have been compromised, as we have demonstrated in Figure 3 and 4. We therefore decideded for a two-step reconstructive approach. Eight months after operation, the patient could speak understandably and the aesthetic result of the soft tissue coverage was satisfactory. Although function of the native tongue can never be reached, the suprafascial ALTP flap was a versatile flap in large defect head and neck reconstruction.
